# Death-Traps

**Published:** 1861-05

**Authors:** 


### DEATH-TRAPS.

Mr. Editor: Those who have, in a measure, lost their health have learned by experience what the unthinking and careless do not know. Strolling through the Central Park, I passed with the crowd into a kind of vault or cave, which was delightfully cool and refreshing; it was provided with seats which were well filled. The temperature within and without was very dissimilar, hence I remained a very short time. On emerging from this retreat, a friend at my side who had not a remembered pain in twenty years, complained of considerable discomfort in drawing in his breath, a fact and a lesson of considerable practical importance, by which the multitudes of merry visitors to our beautiful Central Park, during the coming spring-time and summer, may be warned that in visiting the cave it is best to tarry but for a moment and pass directly on.

I recently visited a Mission Chapel in Sixth avenue, which is well filled with children, twice every Sunday; on any fine day, the windows will be found dropped down, by which the cold air falls immediately on the heads of the little ones. I counted twenty-six persons coughing or sneezing within the hour. In one week the minister reported three deaths from among the children who had assembled there the previous Sabbath. On one occasion, the sexton refused to close the windows at a gentleman's request, who consequently felt obliged to leave the church. Observer.

The remarks of our thoughtful correspondent remind us that the favorite method of airing the rooms of our public-schools is to drop the upper sash two, three, or more inches; the cold air being heavy, falls directly on the heads of the children who are ranged around the wall; and they are compelled to this ordeal daily. It is murderous. We knew a robust, healthy child made sick for a week by a single exposure of the kind; the lower part of the room being warm enough to cause the little thing to remark: "I was almost roasted." It should be impressed on the reader's mind for a life-time, that no air of a room or vehicle, however hot or foul it may be by a crowd and stove-heat, under any ordinary circumstances, is the one hundredth part as pernicious for one hour as a draft of the purest air from the poles for half that time on the occupant who remains still or is a little heated. The very worst that can occur from a crowded omnibus or city car, or from any stove-heated room or church, or other apartment under any common occasion, is a swoon from which the person will recover, perfectly, in a few minutes; but a draft of cold air on a perspiring person for five minutes, or on a person sitting still until chilled, has resulted in life-long maladies, and in death within a week in millions of instances. Better a thousand times faint by foul air and be as well in ten minutes as ever, as will certainly be the case, than by a draft of delicious cool air, have an attack of Pneumonia, of Pleurisy, or some other equally dangerous and fatal disease; for if these ailments do not prove fatal, they always are attended with a very slow recovery; a recovery after months of discomfort oftener than of weeks; and sometimes they leave life-long ailments.

